# Supplementary material for: The nature effect in motion: visual exposure to environmental scenes impacts cognitive load and human gait kinematics
Source: R Soc Open Sci. 2021 Jan 6;8(1):201100. doi: 10.1098/rsos.201100 (PMC7890511; doi:10.1098/rsos.201100)
Supplement: Cognitive motor interference control task [file rsos201100supp1.pdf]

Burtan D, Joyce K, Burn JF, Handy TC, Ho S, Leonards U. 2020
The nature effect in motion: visual exposure to environmental scenes impacts cognitive load and human gait kinematics. R. Soc. Open Sci. 7: 201100. https://doi.org/10.1098/rsos.201100

Supplementary Material Section

1. Cognitive motor interference control task

In line with Ho et al. [1], we established the relationship between cognitive load and gait kinematics as a validation of our 3D motion capture approach. We predicted that gait kinematics collected during walking in our BVI movement laboratory should be sensitive enough to reflect slower and more variable gait with increasing levels of cognitive load, using a verbal dual-task. Participants were the same as in Experiment 1.

1.1. Procedure

Using a repeated measures design, each participant walked repeatedly down a 15m long laboratory whilst completing verbal trail making tasks (vTMT). Dependent on condition, each trial required one of the following vocalisations to be completed: No speech (C1), "Lalala…" (C2), "ABC…" (C3) or "A1B2…" (C4). Note that C3 and C4 are similar to verbal versions of the Trail Making A and B tasks, respectively [2]. Trail making B tasks require a higher amount of cognitive resources than Trail making A tasks as participants have to switch continuously between letters and numbers. The tasks are used in Clinical Neuropsychology to test the limits of cognitive load (e.g. [3]). In our case, the least cognitive resources were required for C1 (no speech; i.e. no dual task requirements, and thus no interference between cognition and walking), and the most for C4.

For each trial, the participant started by standing on a marked cross at one end of the laboratory. The trail condition was then indicated by text projected onto the floor in front of them. When the participant was ready, the text would disappear and the participant walked the length of the laboratory in their natural walking speed whilst carrying out the relevant trail making condition through audible vocalisation. On reaching the end of the laboratory, the participant stopped the verbal task before returning to the starting cross at the other side of the lab. Four practice trials were carried out (1 for each trail type), followed by 20 experimental trials; 5 of each condition, presented in random order. This part of the study took approximately 10 minutes.

Exclusion criteria

One participant was excluded from analysis due to a technical problem with the motion capture system that had resulted in a substantial loss of data. One further participant was excluded from analysis due to having an unusual walking style (mean step length >2.5SD from the group mean) affecting too many trials. This left 18 participants' datasets for analysis; (6 male), aged 18-34 (M = 22). Individual trials were excluded on the basis of missing data (unlabelled markers) or computer errors during the experiment. Moreover, trials in which a participant did not follow the task in the requested way were removed from further analysis. The mean number of trials per condition per participant was 4.92 trials (SD= 0.32) with the inclusion criterion of a minimum of 3 trials.

1.2. Results

We analysed our control task to determine the impact of verbal trail making induced cognitive load on gait for our participant sample. For this, repeated measures MANOVAs were applied to the gait data of the verbal trail making task, with order of experimental parts (Control task; Experiment 1 main task) as a between subjects variable and trail making task condition as a within subjects variable (Cognitive Load; C1/C2/C3/C4) for seven dependent gait measures (gait velocity, mean step length, mean stride time, mean swing time, step length variability, stride time variability and swing time variability).

Note that even though we recorded participants' actual trail making performance to ensure task compliance, we did not include any verbal task performance measures into the analysis.

Velocity: Analysis showed a significant effect of cognitive load on overall velocity (F (3,48) = 20.82; MSE = 0.05; p < 0.001, η_p^2 = 0.57), see Figure 1a. *Post-hoc* tests using Bonferroni correction revealed that participants walked significantly slower during C4 trials (highest cognitive load) than during all other conditions (C1 and C2 p < 0.001, C3 p < 0.01). All other *post-hoc* comparisons yielded insignificant results.

Step Length: Analysis with Greenhouse-Geisser correction showed that there was also a significant main effect of cognitive load on mean step length (F (2.14, 34.28) =11.52; MSE < 0.01; p < 0.001, η_p^2 = 0.42), see Figure 1b. *Post-hoc* tests using Bonferroni correction showed a significantly shorter Step Length for C4 trials (highest cognitive load) as compared to all other conditions (C1 p < 0.05, C2 and C3 p < 0.01). All other *post-hoc* comparisons yielded insignificant results.

Stride Time: Analysis with Greenhouse-Geisser correction showed that there was a significant main effect of cognitive load on mean stride time (F (1.97, 31.51) = 17.24; MSE < 0.001; p < 0.001, η_p^2 = 0.52), see Figure 1c. *Post-hoc* tests using Bonferroni correction revealed significantly longer stride times for C4 trials (highest cognitive load) as compared to all other conditions (C1 and C2 p < 0.01, C3 p < 0.05). Moreover, stride times for C3 trials were also significantly longer than for C1 trials (p < 0.01) and C2 trials (p < 0.05).

Swing Time: There was a significant main effect of cognitive load on mean swing time (F (3,48) =6.42; MSE < 0.001; p < 0.01, η_p^2 = 0.29), see Figure 1d. *Post-hoc* tests using Bonferroni correction showed a significantly longer swing time for C4 trials (highest cognitive load) as compared to C1 (p < 0.05). Moreover, swing times for C3 trials were also significantly longer than for C1 trials (p < 0.05).

There was no main effect of cognitive load on the variability of step length, the variability of stride time and the variability of swing time.

There was no effect of experimental part order on any of the seven dependent measures. However, there was a significant interaction between cognitive load and part order for swing time (F (3,48) = 3.36; MSE p< 0.001; p < 0.05, η_p^2 = 0.17).

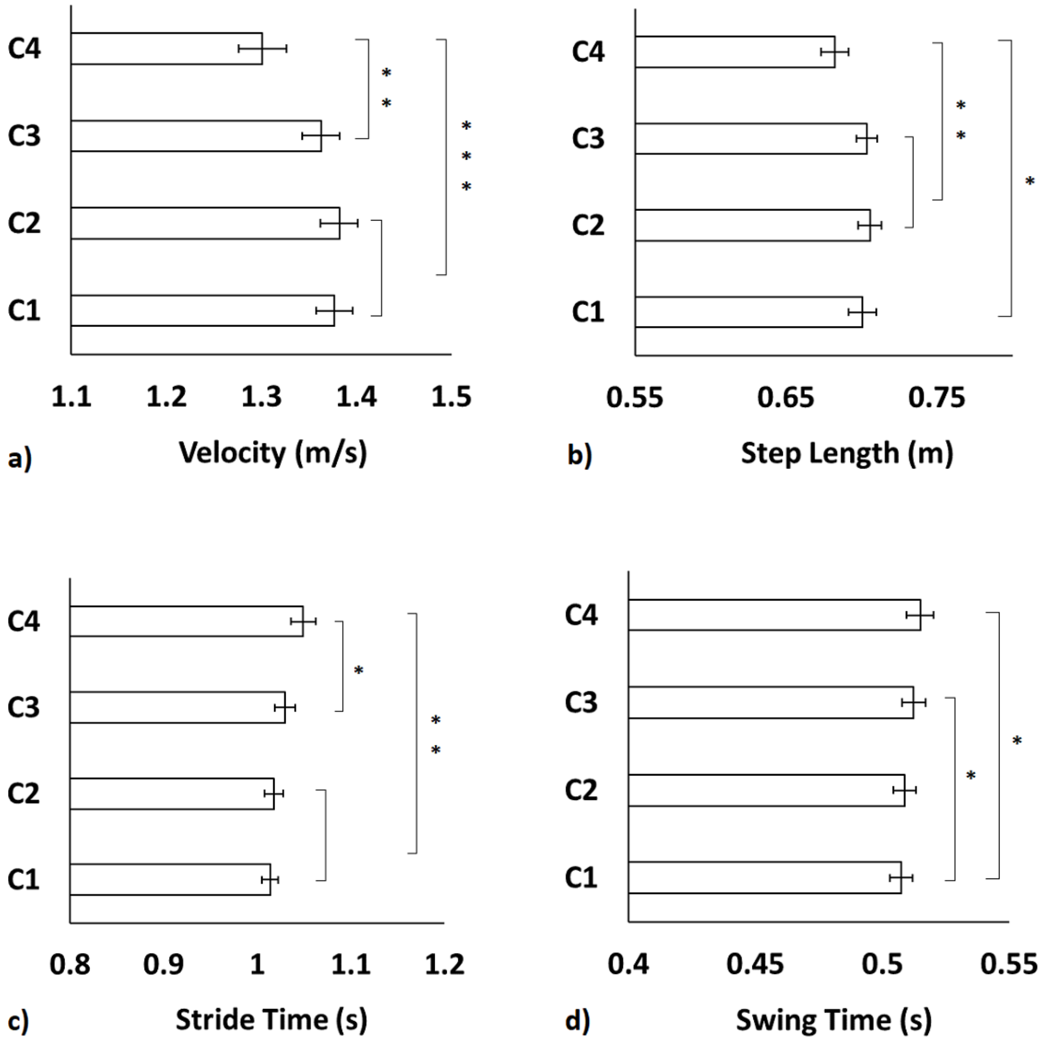

*Fig 1: Group averages of a) mean velocity (in m/s), b) step length (in meters), c) stride time (in seconds) and d) swing time (in seconds) across varying levels of cognitive load. C1 = no speech, C2 = lalala, C3 = ABC, C4 = A1B2. Error bars reflect ±1SEM. * p < 0.05, ** p < 0.01, *** p < 0.001.*

Overall, these results confirm once more that increases in cognitive load can be reliably measured in young healthy participants, using gait kinematics, in particular step size and overall gait velocity in line with earlier studies by Ho et al.[1] and Kannape et al.[4]. Moreover, when compared to our main study (Experiment 1), results also confirm our expectations that changes in cognitive load introduced by differences in visual environments are far more subtle than those produced by the most cognitively demanding verbal task (C4) used here.

Interaction between cognitive load and part order for swing time in a cognitive motor interference control task

See Table 1 for Group averages of mean swing time (in seconds) across varying levels of cognitive load and order.

	Order 1		Order 2	
Swing Time	M	SD	M	SD
C1	0.51	0.02	0.51	0.02
C2	0.51	0.02	0.51	0.02
C3	0.51	0.02	0.52	0.02
C4	0.51	0.02	0.52	0.02

Note. C1 = no speech, C2 = lalala, C3 = ABC, C4 = A1B2. Order 1= cognitive motor interference control task as the first task and environmentally induced perceptual load – motor interference task as the second task; N = 9, Order 2 = environmentally induced perceptual load – motor interference task as the first task and cognitive motor interference control task as the second task; N = 9.

Interaction between environment and part order for velocity and stride time in environmentally induced perceptual load – motor interference task

See Table 2 for group averages of mean velocity (m/s) + standard deviations across environment type and order

	Order 1		Order 2	
Velocity	M	SD	M	SD
Nature	1.30	0.07	1.30	0.06
Urban	1.29	0.07	1.28	0.06
Neutral	1.33	0.07	1.36	0.06

Note. Order 1 = cognitive motor interference control task as the first task and environmentally induced perceptual load – motor interference task as the second task; N = 10, Order 2 = environmentally induced perceptual load – motor interference task as the first task and cognitive motor interference control task as the second task; N = 8.

See Table 3 for Group averages of mean stride time (in seconds) and standard deviations across environment type and order

	Order 1		Order 2	
Stride Time	M	SD	M	SD
Nature	1.04	0.04	1.04	0.04
Urban	1.04	0.04	1.05	0.04
Neutral	1.03	0.03	1.02	0.03

Note. Order 1 = cognitive motor interference control task as the first task and environmentally induced perceptual load – motor interference task as the second task; N = 10, Order 2 =

environmentally induced perceptual load – motor interference task as the first task and cognitive motor interference control task as the second task; N = 8.

References

1. Ho S., Mohtadi A., Daud K., Leonards U., Handy T.C. 2019 Using smartphone accelerometry to assess the relationship between cognitive load and gait dynamics during outdoor walking. *Sci Rep* **9**(1), 3119. (doi:10.1038/s41598-019-39718-w).
2. Bowie C.R., Harvey P.D. 2006 Administration and interpretation of the Trail Making Test. *Nat Protoc* **1**(5), 2277-2281. (doi:10.1038/nprot.2006.390).
3. Nasreddine Z.S., Phillips N.A., Bedirian V., Charbonneau S., Whitehead V., Collin I., Cummings J.L., Chertkow H. 2005 The Montreal Cognitive Assessment, MoCA: a brief screening tool for mild cognitive impairment. *J Am Geriatr Soc* **53**(4), 695-699. (doi:10.1111/j.1532-5415.2005.53221.x).
4. Kannape O.A., Barre A., Aminian K., Blanke O. 2014 Cognitive loading affects motor awareness and movement kinematics but not locomotor trajectories during goal-directed walking in a virtual reality environment. *PLoS One* **9**(1), e85560. (doi:10.1371/journal.pone.0085560).
